# Optical coherence tomography angiography helps distinguish multiple sclerosis from AQP4‐IgG‐seropositive neuromyelitis optica spectrum disorder

**DOI:** 10.1002/brb3.2125

**Published:** 2021-03-30

**Authors:** Chunxin Liu, Hui Xiao, Xiayin Zhang, Yipeng Zhao, Rui Li, Xiaonan Zhong, Yuge Wang, Yaqing Shu, Yanyu Chang, Jingqi Wang, Caixia Li, Haotian Lin, Wei Qiu

**Affiliations:** ^1^ Department of Neurology Third Affiliated Hospital Sun Yat‐sen University Guangzhou China; ^2^ Zhongshan Ophthalmic Center Sun Yat‐sen University Guangzhou China; ^3^ School of Mathematics Sun Yat‐sen University Guangzhou China

**Keywords:** multiple sclerosis, neuromyelitis optica spectrum disorder, optical coherence tomography angiography

## Abstract

**Introduction:**

The aim was to characterize the optical coherence tomography (OCT) angiography measures in patients with multiple sclerosis (MS) and neuromyelitis optica spectrum disorder (NMOSD) and to evaluate their disease discrimination capacity.

**Methods:**

Patients with MS (*n* = 83) and AQP4‐IgG‐seropositive NMOSD (*n* = 91) with or without a history of optic neuritis, together with healthy controls (*n* = 34), were imaged. The main outcome measures were peripapillary retinal nerve fiber layer (pRNFL) thickness, macular ganglion cell‐inner plexiform layer (GC‐IPL) thickness, macular vessel density (VD), and perfusion density (PD) in the superficial capillary plexus. Diagnostic accuracy was assessed using the area under the receiver operating characteristics curve.

**Results:**

Compared with patients with MS, those with NMOSD had a significantly smaller average thickness of the pRNFL and GC‐IPL (80.0 [59.0; 95.8] μm versus 92.0 [80.2; 101] μm, *p* < .001; 68.0 [56.0; 81.0] μm, versus 74.5 [64.2; 81.0] μm, *p* < .001) and significantly smaller whole VD and PD areas (15.6 [12.6; 17.0] mm^−1^ versus 16.7 [14.8; 17.7] mm^−1^, *p* < .001; 0.38 [0.31; 0.42] mm^−1^ versus 0.40 [0.37; 0.43] mm^−1^, *p* < .01). The combination of structural parameters (average thickness of the pRNFL and GC‐IPL) with microvascular parameters (temporal‐inner quadrant of VD, temporal‐inner, nasal‐inferior, and nasal‐outer quadrant of PD) was revealed to have a good diagnostic capability for discriminating between NMOSD and MS.

**Conclusions:**

OCT angiography reveals different structural and microvascular retinal changes in MS and AQP4‐IgG‐seropositive NMOSD. These combined structural and microvascular parameters might be promising biomarkers for disease diagnosis.

## Graphical abstract

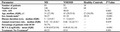

## 1 INTRODUCTION

Multiple sclerosis (MS) and neuromyelitis optica spectrum disorder (NMOSD) are two major idiopathic inflammatory demyelinating diseases of the central nervous system (CNS) causing non‐traumatic neurologic disability among young individuals (Browne et al., 2014; Weinshenker & Wingerchuk, 2017). In addition to intracranial and spinal cord lesions, both diseases are associated with a poor visual prognosis as patients often suffer from recurrent episodes of optic neuritis (ON). (Jeong et al., 2016; Kwapong et al., 2018) AQP4‐IgG‐seropositivity is detected in up to 80% of patients who meet the clinical and radiologic criteria for NMOSD. (Jarius et al., 2020) The overlapped clinical manifestations of both diseases make early precise diagnosis difficult. Indeed, MS and NMOSD have different pathophysiological mechanisms, recommended treatment strategies for attack prevention, and prognoses (Akaishi et al., 2020; Zubizarreta et al., 2019). Being able to assess the risk of progression or the level of CNS pathology using readily measurable biomarkers would be of great value.

The retina and brain share common embryological origins and vascular supply. There is ample evidence that the retina provides an accurate window into the brain (Calabresi et al., 2010). Optical coherence tomography (OCT) techniques noninvasively provide quantitative information on optic nerve structure, with the advantages of short acquisition times and sensitive measurements (Ohayon et al., 2020; Spain et al., 2018). OCT‐derived retinal layer volumes correlate with both brain and spinal cord volumes, suggesting that OCT serves as a tool for the evaluation of neurodegeneration in the course of MS and NMOSD (Oh et al., 2015; Saidha et al., 2013). Notably, a recent study combining OCT and multifocal visual evoked potential provided clinical evidence supporting the hypothesis that progressive axonal damage is associated with chronic demyelination in MS. (You et al., 2020) In addition, retinal atrophy is present in the absence of ON. Foveal morphometry described a flatter and wider fovea in the NMOSD group in comparison with both the MS and healthy control (HC) groups, suggesting that these changes cannot be explained by neuroaxonal damage resulting from ON alone (Motamedi et al., 2020). In this context, OCT measurements can provide key information for differential diagnoses and for disease course monitoring in the clinical trials of neuroinflammatory disease (Oertel et al., 2018; Tavazzi et al., 2020). The most common retinal abnormality during OCT assessments of the MS and NMOSD groups is a thinning of the peripapillary retinal nerve fiber layer (pRNFL) and the macular ganglion cell‐inner plexiform layer (GC‐IPL). (Bennett et al., 2015; Fernandes et al., 2013; Filippatou et al., 2020; Lange et al., 2013; Merle et al., 2008).

OCT angiography (OCTA) represents a novel noninvasive imaging technique derived from additional processing of OCT. It generates high‐resolution information of retinal blood vessels and retinal vascular flow and is likely to provide more useful information on MS and NMOSD. This method of assessing cerebral disease processes has been applied to other CNS disorders (Alber et al., 2020).

While the pRNFL comprises axons originating from ganglion cell neurons and reveals the optic nerve status, the macular GC‐IPL is a direct reflection of the intrinsic ganglion cell bodies that can provide information on primary retinal pathology. (Gabilondo et al., 2015; Jeong et al., 2016; Rebolleda et al., 2015) There is little evidence supporting the use of OCTA in MS and NMOSD eyes; thus, we used OCTA to provide a descriptive and detailed overview of alterations in the macular structure and microvasculature and to compare the characteristics in MS with those in NMOSD.

## 2 METHODS

### 2.1 Patient recruitment

In this retrospective study, participants were recruited from the Department of Neurology of the Third Affiliated Hospital and the Zhongshan Ophthalmic Center, Sun Yat‐sen University, between April 2019 and May 2020. Written informed consent was obtained from all participants. The study was approved by the Ethics Committee of the Third Affiliated Hospital, Sun Yat‐sen University, and was conducted in accordance with the tenets of the Declaration of Helsinki.

The exclusion criteria were as follows: (a) diagnosis of other systemic diseases, including hypertension or diabetes; (b) ocular diseases, including glaucoma, cataract, myopia (<−6 diopter), or hyperopia (>6 diopter), or eye surgeries; (c) age < 18 years; and (d) inability to provide informed consent. The enrolled patients were diagnosed with MS and NMOSD by a neurologist (Wei Qiu, M.D., Ph.D.) based on the 2017 McDonald Criteria and the 2015 IPND criteria, respectively. (Thompson et al., 2018; Wingerchuk et al., 2015) Patients with an episode of ON within the last 6 months were excluded to minimize the effect of optic disc swelling. HCs were recruited from the same hospital staff. Patients were included in the HC group if they had no family history of MS or NMOSD, normal‐appearing optic disc and normal visual acuity.

### 2.2 Clinical assessments

All participants underwent an extensive ophthalmologic examination performed by a well‐trained clinician, including assessments of best‐corrected visual acuity (BCVA) using the logarithm of the minimum angle of resolution unit, slit‐lamp biomicroscopy, fundus photography, and OCTA. Meanwhile, all participants underwent a clinical neurological examination with assessment of the Expanded Disability Status Scale score on the same day. Anti‐AQP4‐immunoglobulin G (IgG) and anti‐MOG‐IgG were determined using a fixed cell‐based assay indirect immunofluorescence test (Euroimmun AG, Lübeck Germany). To minimize heterogeneity, we only included NMOSD patients who were AQP4‐IgG seropositive and MOG‐IgG negative and MS patients who were seronegative both for AQP4‐IgG and MOG‐IgG. A history of ON was defined as an acute relapse lasting more than 24 hr with high‐contrast vision acuity loss, eye movement pain, color vision impairments, or optic nerve enhancement as shown on MRI.

### 2.3 OCTA acquisition and processing

**FIGURE 1:**
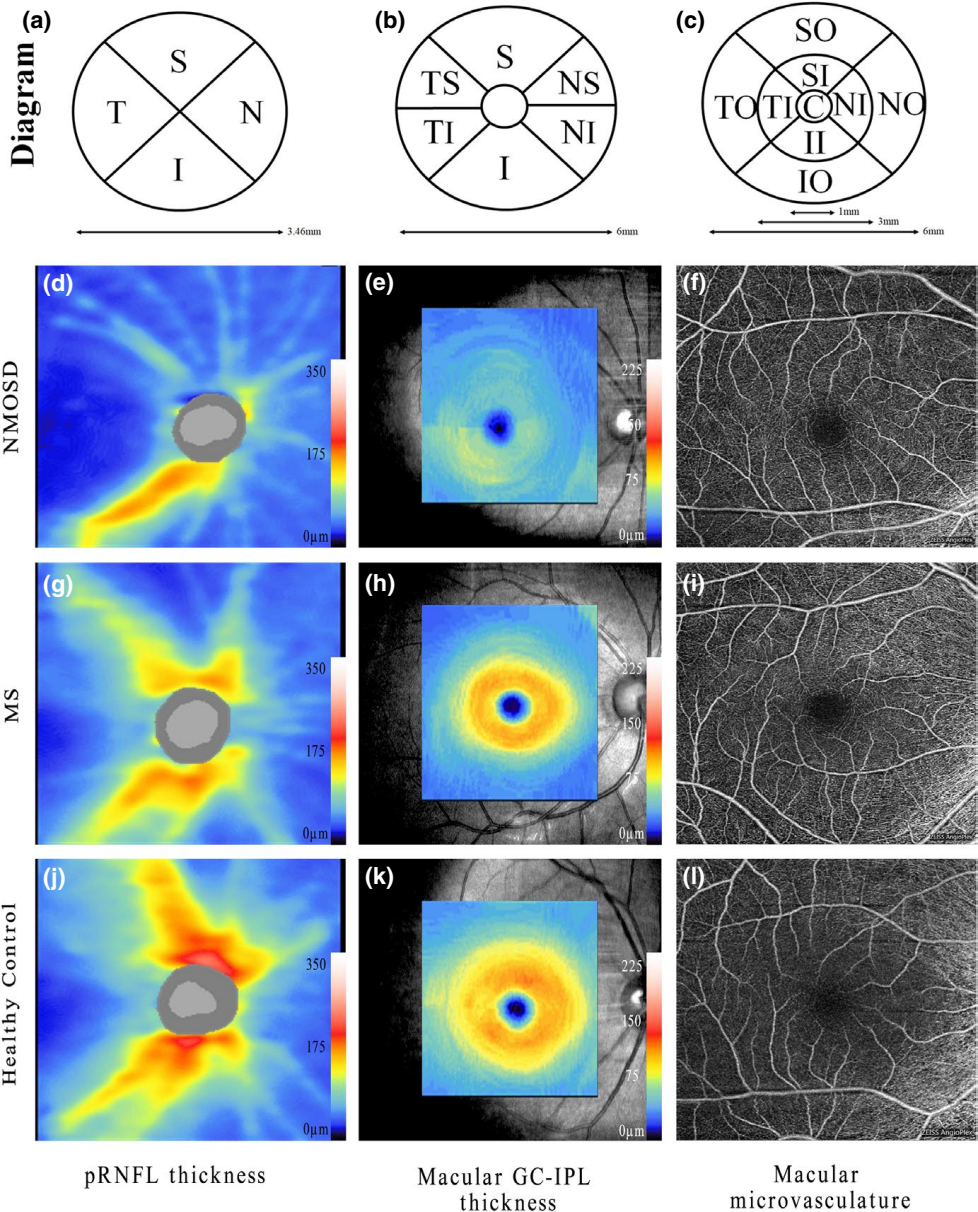
Structural and microvasculature measurements in representative eyes. (A, B, C) Diagram of macular and peripapillary measurements using optical coherence tomography angiography. pRNFL thickness was divided into four sectors, macular GC‐IPL thickness was divided into six sectors and macular microvasculature was divided into nine sectors. OCTA images of pRNFL thickness, GC‐IPL thickness, and macular microvasculature: (i) in an eye from a patient with NMOSD (D, E, F); (ii) in an eye from a patient with MS (G, H, I); and (iii) in an eye from a healthy control (J, K, L.). Macular microvasculature was measured in the superficial capillary plexus. NMOSD: neuromyelitis optica spectrum disorder; MS: multiple sclerosis; pRNFL: peripapillary retinal nerve fibre layer; GC‐IPL, GC‐IPL; S: superior; I: inferior; N: nasal; NI: nasal‐inferior; NS: nasal‐superior; T: temporal; TI: temporal‐inferior; TS: temporal‐superior; SO: superior‐outer; TO: temporal‐outer; IO: inferior‐outer; NO: nasal‐outer; SI: superior‐inner; TI: temporal‐inner; II: inferior‐inner; NI: nasal‐inner; C: central

OCTA imaging was performed using an ultra‐clear OCT and AngioPlex device (Cirrus 5,000, version 10.0; Zeiss Meditec, California, USA) as described previously. (Zhang, Xiao, Liu, Liu, et al., 2020; Zhang, Xiao, Liu, Zhao, et al., 2020) Briefly, we selected an optic disc cube 200 × 200 scan protocol for pRNFL measurements. pRNFL thickness was measured within 3.46‐mm‐diameter circles around the optic disc. The average and the thickness of four quadrant sectors (superior, temporal, inferior, and nasal) were assessed. The ganglion cell analysis algorithm was used to process the data obtained with the macular cube 512 × 128 scan protocol to calculate the macular GC‐IPL thickness within a 14.13‐mm^2^ elliptical annular area. The average thickness and six sectors (superior, temporal‐superior, temporal‐inferior, inferior, nasal‐inferior, and nasal‐superior) thickness were analyzed. Angiography imaging was performed centered at the macula with a 6 × 6‐mm scan pattern. Vessel density (VD) was calculated from the total length of the perfused vasculature per unit area in a region of measurement, while perfusion density (PD) corresponded to the total area of perfused vasculature per unit area in the region of measurement. The VD and PD values of the whole area and nine quadrant sectors (central, superior‐inner, temporal‐inner, inferior‐inner, nasal‐inner, superior‐outer, temporal‐outer, inferior‐outer and nasal‐outer) were analyzed. VD and PD measurements were performed on the annular zone after exclusion of the foveal avascular zone (FAZ). Structural and microvascular measurements in representative eyes are shown in Figure 1. Images with a scan quality <7 or with residual motion artifacts were excluded from data analysis. All images were assessed in a blinded fashion by experienced reviewers (L.C.X and Z.X.Y), and all OCT scans were quality‐controlled according to the OSCAR‐IB criteria. (Schippling et al., 2015) OCT data were consistent with the respected Advised Protocol for OCT Study Terminology and Elements recommendations. (Cruz‐Herranz et al., 2016).

### 2.4 Statistical analyses

All statistical analyses were performed using software R, version 3.4 (Institute for Statistics and Mathematics, www.r‐project.org). Comparisons were adjusted for the following prespecified confounding factors: age, sex, and the inter‐eye correlation in each participant. OCT measures were compared between groups with the generalized estimating equations (GEE) models using the “geeglm” function of the package, “geepack”, (J, 2002) thereby accounting for within‐subject inter‐eye correlations. Pearson's correlations and bootstrapped *p*‐values were used to assess associations between the OCT parameters and correlations between OCTA parameters and BCVA. The area under the receiver operating characteristic curve (AUC) was used to calculate the diagnostic power of the logistic models. The AUC values were compared using the method described by DeLong et al. (DeLong et al., 1988) Statistical significance was set to *p* < .05.

## 3 RESULTS

### 3.1 Summary of demographic and clinical data

After exclusion of poor‐quality OCTA images, 83 patients with MS (146 eyes; 76 with ON and 70 without ON), 91 patients with AQP4‐IgG‐seropositive NMOSD (135 eyes; 90 with ON and 45 without ON), and 34 HCs (68 eyes) were enrolled. Among patients with MS, disease‐modifying therapies (DMTs) used included teriflunomide in 34 (41%), rituximab in 11 (13.3%), fingolimod in 4 (4.8%), interferon‐α in 1 (1.2%), and dimethyl fumarate in 1 (1.2%) patient. Traditional immunosuppressants were also used, including azathioprine (6 [7.2%] patients), mycophenolate (1 [1.2%] patient), and tacrolimus (2 [2.4%] patients). Twenty‐three (27.7%) patients were not using DMTs. Among patients with NMOSD, the immunosuppressants used were azathioprine (33 [36.3%] patients), mycophenolate (44 [48.4%] patients), and rituximab (6 [6.6%] patients). Eight (8.7%) patients were not using immunosuppressants (Table 1).

**TABLE 1:** Demographic data and clinical characteristics of patients

Parameter	MS	NMOSD	Healthy Controls	*P* Value
Number of patients	83	91	34	—
Number of eyes	146	135	68	—
+ON: −ON	76:70	90:45	—	0.003
Age, median, (IQR), y^a^	31 (27;38)	41 (29;53.8)	27 (24;29)	<0.001
Sex, female: male^b^	56:27	81:10	20:14	<0.001
Disease duration (yrs), median (IQR)	5 (2;9.45)	5 (2.6;8.9)	—	0.249
Annual recurrence rate, median (IQR)	0.52 (0.1;0.82)	0.46 (0.2;0.71)	—	0.659
Percentage of patients with OCB+	54.7%	—	—	—
BCVA (LogMAR), mean (IQR)	0.30 (0.07;0.54)	0.40 (0.18;1.0)	—	<0.001
Treatment: No‐Treatment^c^	60:23	83:8	—	0.001

Note

BCVA, best‐corrected visual acuity; EDSS, Expanded Disability Status Scale; LogMAR, logarithm of minimum angle of resolution;

^a^ Kruskal–Wallis test, for comparisons between three groups, *P*‐value < 0.05 was considered to be statistically significant.

^b^ Chi‐square test, for comparisons between three groups, *P*‐value < 0.05 was considered to be statistically significant.

^c^ Treatment: No‐Treatment, for MS DMT: no‐DMT; for NMOSD Immunosuppressants: No‐ Immunosuppressants.

### 3.2 Comparison of OCT structural parameters and OCTA microvascular parameters in MS, NMOSD, and HC groups

**FIGURE 2:**
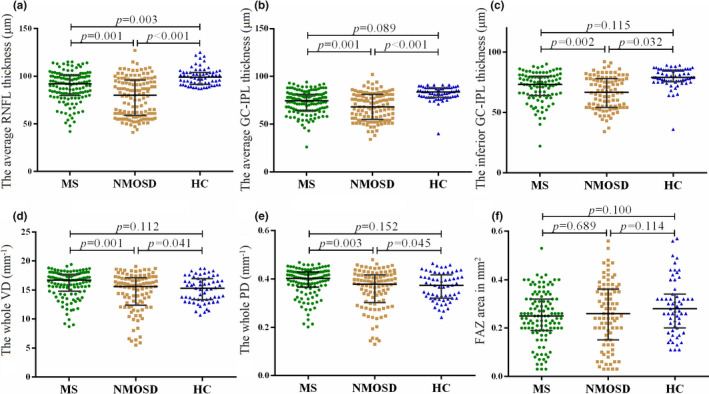
Patterns of losses in macular structural and microvasculature. (A‐E) The NMOSD group showed significant thinning of average pRNFL, average GC‐IPL, inferior GC‐IPL, whole VD area, and whole PD area compared to MS group. (F) The FAZ area showed no significant correlation between MS group and NMOSD group

The average pRNFL thickness in MS and NMOSD groups was remarkably lower than that in the HC group (MS 92.0 [80.2; 101] μm, NMOSD 80.0 [59.0;95.8] μm, versus HCs: 99.0 [92.0; 104] μm, *p* < .001). The average GC‐IPL thickness in MS and NMOSD groups were significantly lower than that in the HC group (MS 74.5 [64.2; 81.0], NMOSD 68.0 [56.0; 81.0] versus HCs 83.5 [78.0; 88.0] μm, *p* < .001). However, there was no significant difference between MS or NMOSD groups and the HCs in VD and PD areas. No significant difference in FAZ was found in a comparison between the three groups (Figure 2, Appendix Table 1).

### 3.3 pRNFL and GC‐IPL thicknesses in subgroups

**FIGURE 3:**
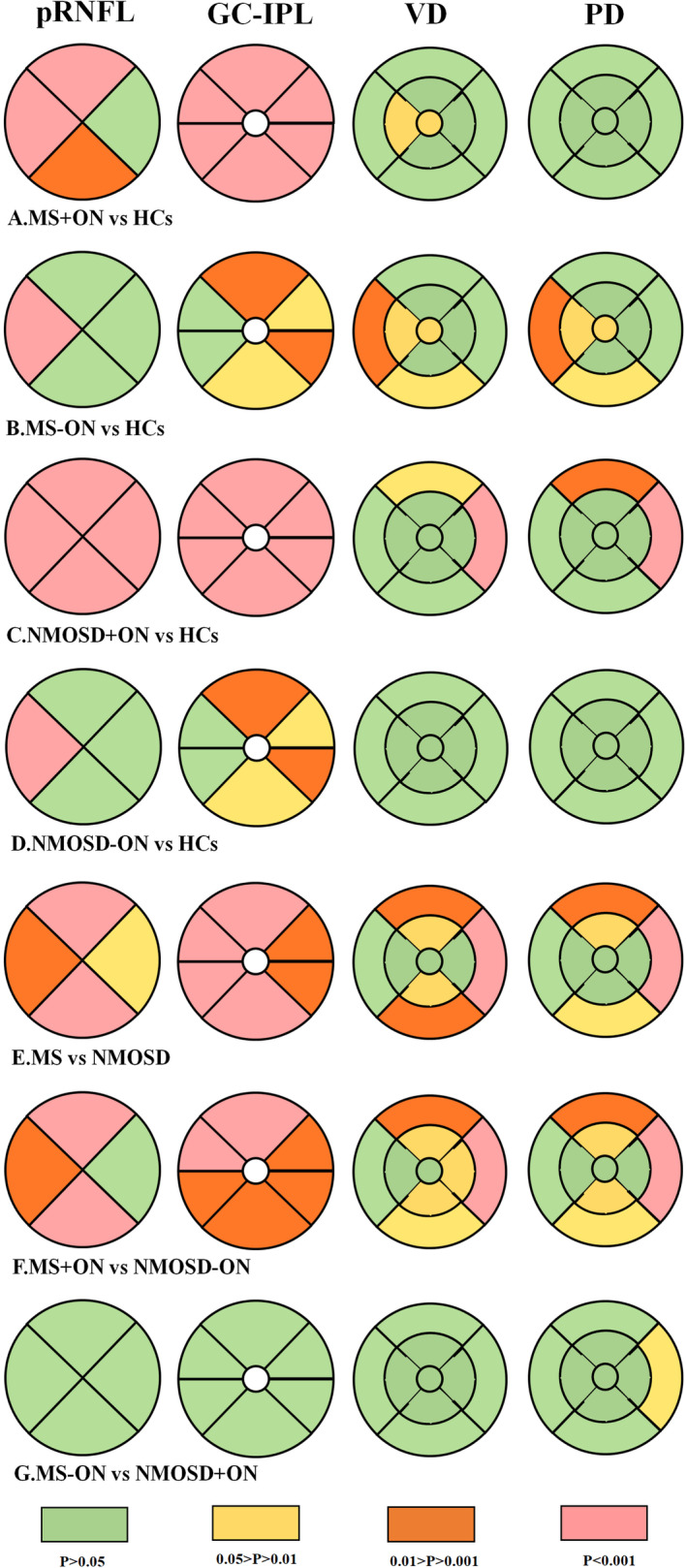
Comparison of structural and angiography parameters between groups. After adjusting for age and sex, the overall *P*‐values of the five groups (HCs, MS‐ON, MS + ON, NMOSD‐ON, NMOSD + ON) were smaller than 0.05 (by GEE method). The latter subgroup comparison is pairwise comparison of *P*‐values by Hochberg method. (A–D) Topographic damages of MS + ON, MS‐ON, NMOSD + ON, and NMOSD‐ON compared with healthy controls. (E) The thickness of the pRNFL and GC‐IPL was significantly reduced in the inferior and superior quadrants in NMOSD than in MS. The NMOSD group showed significantly reduced macular VD compared to the MS group in superior‐outer, nasal‐outer, and inferior‐outer quadrants. The NMOSD group showed significantly reduced macular PD compared to the MS group in superior‐outer, inferior‐outer, and nasal‐outer quadrants. (F) The thickness of the pRNFL and GC‐IPL were significantly reduced in the superior and inferior quadrants in NMOSD + ON, and NMOSD + ON group showed significantly reduced macular VD and PD compared to MS + ON in superior‐outer quadrant. (G) Topographic damages of MS‐ON and NMOSD‐ON were pretty much the same in all aspects except in nasal‐outer quadrants PD

The NMOSD group showed a significantly smaller average pRNFL thickness than the MS group (80.0 [59.0; 95.8] μm versus 92.0 [80.2; 101] μm, *p* < .001). The pRNFL thickness at the six quadrants was also comparable between the MS and NMOSD groups (*p* < .001). The comparison of average GC‐IPL thicknesses between MS and NMOSD patients was remarkable (74.5 [64.2; 81.0] μm, versus 68.0 [56.0; 81.0] μm, *p* < .001), and a significant difference was observed in all six quadrants. The average quadrants’ pRNFL thickness values were also comparable between the MS + ON and NMOSD + ON subgroups (83.0 [71.0; 92.0] μm versus 63.0 [55.0; 84.0] μm, *p* < .001), and a significant difference was observed in all quadrants except for the nasal quadrant. There was also a significant difference between the two subgroups in all quadrants in terms of GC‐IPL thickness. The MS‐ON and NMOSD‐ON subgroups showed no significant differences in the average pRNFL or GC‐IPL thicknesses compared with the HCs (Figure 3, Appendix Table 2).

### 3.4 VD and PD areas and FAZ in subgroups

The NMOSD group showed dramatically smaller whole VD and PD areas than the MS group (15.6 [12.6; 17.0] mm^−1^ versus 16.7 [14.8; 17.7] mm^−1^, *p* < .001; 0.38 [0.31; 0.42] mm^−1^ versus 0.40 [0.37; 0.43] mm^−1^, *p* < .01).

Compared with the MS group, the NMOSD group showed a significantly smaller macular VD area in the superior‐inner, inferior‐inner, superior‐outer, inferior‐outer, and nasal‐outer quadrants (16.0 [13.1; 17.5] mm^−1^ versus 16.9 [14.6; 18.0] mm^−1^, 15.4 [12.5; 17.4] mm^−1^ versus 16.1 [13.9; 17.6] mm^−1^, 16.3 [13.2; 17.7] mm^−1^ versus 17.3 [15.9; 18.3] mm^−1^, 15.6 [13.0; 17.4] mm^−1^ versus 16.4 [14.6; 17.9] mm^−1^, 17.9 [14.3;19.3] mm^−1^ versus 19.4 [17.7; 19.9], *p* < .05, respectively) and a significantly smaller macular PD area in the superior‐inner, superior‐outer, inferior‐outer, and nasal‐outer quadrants (0.38 [0.30; 0.43] mm^−1^ versus 0.40 [0.35; 0.43] mm^−1^, 0.40 [0.33; 0.44] mm^−1^ versus 0.43 [0.39; 0.46] mm^−1^, 0.38 [0.32; 0.43] mm^−1^ versus 0.41 [0.35; 0.44] mm^−1^, 0.44 [0.35; 0.47] mm^−1^ versus 0.47 [0.44; 0.49] mm^−1^, *p* < .05, respectively).

The macular VD and PD areas in the superior‐inner, inferior‐inner, superior‐outer, inferior‐outer, and nasal‐outer quadrants were significantly smaller in the NMOSD + ON than in the MS + ON subgroup. No significance of FAZ was found between the patient subgroups (Figure 3, Appendix Tables 1 and 2).

### 3.5 Correlation analysis between structural and microvascular parameters

The whole VD area was correlated with the average pRNFL thicknesses in the disease groups (MS: *R* = 0.25, *p* < .01; NMOSD: *R* = 0.36, *p* < .001; HCs: *R* = 0.15, *p* = .26).

The whole VD area was correlated with the average GC‐IPL thicknesses in the disease groups (MS: *R* = 0.29, *p* < .01; NMOSD: *R* = 0.41, *p* < .001; HCs: *R* = 0.16, *p* = .26).

The whole PD area was significantly correlated with the average pRNFL thicknesses in the disease groups (MS: *R* = 0.27, *p* < .01; NMOSD: *R* = 0.39, *p* < .001; HCs: *R* = 0.17, *p* = .19).

The whole PD area was significantly correlated with the average GC‐IPL thicknesses in the disease groups (MS: *R* = 0.31, *p* < .001; NMOSD: *R* = 0.45, *p* < .001; HCs: *R* = 0.18, *p* = .18) (Appendix Figure 1).

### 3.6 Correlation analysis between OCTA values and visual function

In the MS + ON group, there were no significant relationship between the BCVA, the average pRNFL thickness and the average GC‐IPL thickness. In the NMOSD + ON group, the BCVA was significantly correlated with the average pRNFL thickness (*r* = −0.48, *p* < .001), but no significant correlation with the average GC‐IPL thickness (*R* = −0.22, *p* = .065). No statistically significant correlations were found between the BCVA and the whole VD and PD in either MS or NMOSD patients (Appendix Figure 2).

### 3.7 Diagnostic accuracy of OCTA parameters

**FIGURE 4:**
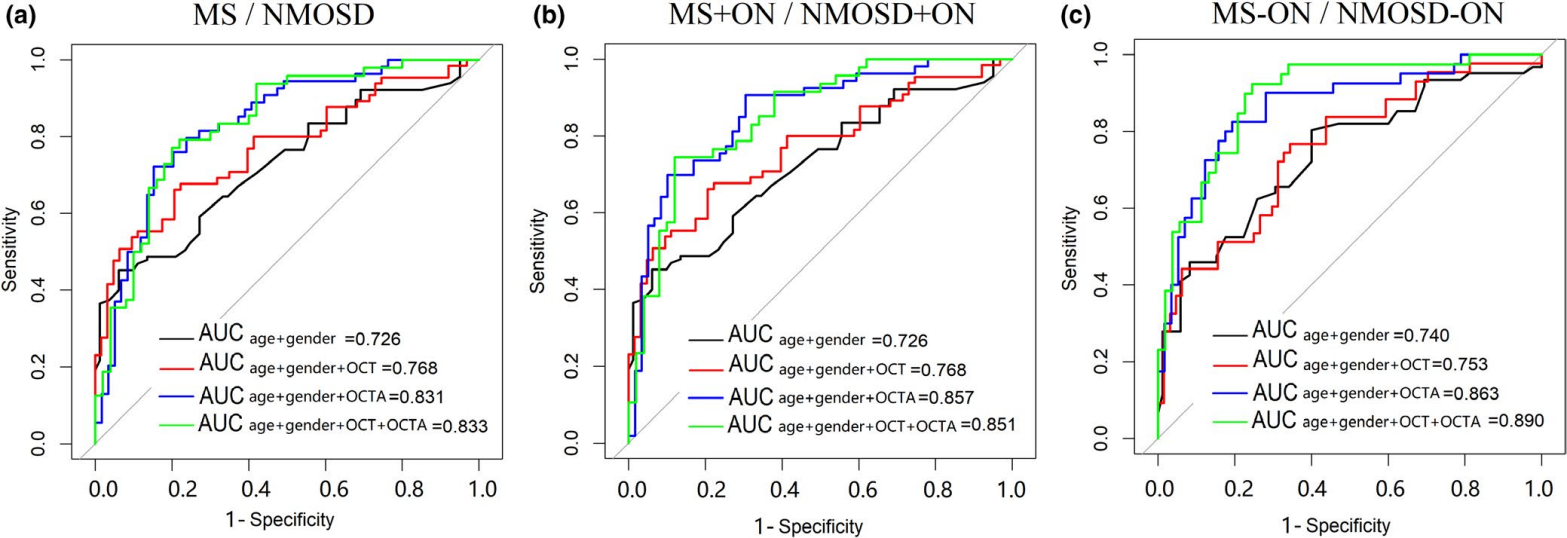
Diagnostic accuracies of OCTA parameters in discriminating among patients with MS, patients with NMOSD. (A) Distinguishing MS and NMOSD from each other. In the specific structural OCT parameters, average pRNFL, and GC‐IPL were selected combine with (TI quadrant of VD and PD, NI and NO quadrant of PD) showed the best diagnostic capability were selected in the OCTA parameters. (B) OCTA parameters (IO and NO quadrant of VD and PD) showed the best diagnostic capability for the discrimination from MS + ON and NMOSD + ON. (C) For the detection of MS‐ON/NMOSD‐ON, combined OCT parameters (average pRNFL, average GC‐IPL) and OCTA parameters (IO and NO quadrant of VD and PD) showed the best diagnostic capability for the discrimination. MS: multiple sclerosis; NMOSD: neuromyelitis optica spectrum disorder; ON: optic neuritis; OCTA: optical coherence tomography angiography; pRNFL: peripapillary retinal nerve fiber layer; GC‐IPL, GC‐IPL; S: superior; I: inferior; N: nasal; NI: nasal‐inferior; NS: nasal‐superior; T: temporal; TI: temporal‐inferior; TS: temporal‐superior; VD: vessel density; PD: perfusion density

To discriminate MS from NMOSD, the structural OCT (average pRNFL thickness and average GC‐IPL thickness) combined with the OCTA parameters (temporal‐inner quadrants of VD, temporal‐inner, nasal‐inferior, and nasal‐outer quadrants of PD) showed the best diagnostic capability (AUC 0.833). For the detection of MS + ON/NMOSD + ON, we quantified the diagnostic capabilities of the OCTA parameters and found that the inferior‐outer and nasal‐outer quadrants of VD and PD showed the best degree of discrimination in terms of diagnostic capability (AUC 0.857) (Figure 4, Appendix Table 3).

## 4 DISCUSSION

In this study, we characterized the retinal structural and microvascular changes in MS and AQP4‐IgG positive NMOSD eyes with/without a history of ON and analyzed their disease differentiation ability. Compared to patients with MS, patients with NMOSD showed a significantly smaller average thickness of pRNFL and GC‐IPL, and significantly smaller whole areas of VD and PD. The combination of structural parameters with microvascular parameters showed the best diagnostic capability for discriminating between these two diseases.

MS and NMOSD are both autoimmune demyelinating diseases of the CNS. Specifically, MS is a disease mainly affecting myelin and oligodendrocytes, whereas NMOSD is primarily an autoimmune astrocytopathy. They have two pathogenic components, namely, inflammation and neurodegeneration, but different degrees of severity and pathogenetic mechanisms. Thus, they result in distinct epidemiology, immunopathogeneses, diagnoses, and treatment features. (Jarius et al., 2020; Kawachi & Lassmann, 2017) Abnormalities occurring within the brain could be reflected in the retina and are, therefore, quantifiable with high‐resolution OCT imaging modalities. (Ayrignac et al., 2014; Dogan et al., 2019; Hokari et al., 2016; Shen et al., 2019) In this context, OCT can provide further insights into the pathogenesis by a direct comparison between these two diseases. Nowadays, vascular changes are increasingly recognized as important factors in the pathophysiology of neuroinflammatory diseases, including MS and NMOSD. Compared with traditional OCT metrics, the novel technology of OCTA is also noninvasive. Additionally, it images retinal vascular density in a depth‐resolved manner and provides an alternative quantitative measure of retinal damage with less “floor‐effect”. (Kleerekooper et al., 2020; Oertel et al., 2017) To the best of our knowledge, this is the first study to use OCT and OCTA metrics to assess the patterns of retinal structural and microvasculature changes in patients with MS and NMOSD.

Our study indicated that the patterns and severity of macular structure and microvasculature loss differed significantly between MS and NMOSD. pRNFL and GC‐IPL thinning were more severe in NMOSD patients, particularly in the superior and inferior quadrants and average segments. This result was consistent with a recent meta‐analysis study. (Filippatou et al., 2020) Patients with NMOSD had significantly smaller whole VD and PD areas than those with MS. A recent study showed that NMOSD + ON eyes were associated with worse visual outcomes than MS + ON eyes, even with similar severity of their macular GC‐IPL thinning. (Sotirchos et al., 2020) Our results confirm this finding. The results of our study further demonstrate that the degree and patterns of changes in MS and NMOSD reflect the different pathophysiological mechanisms that underlie these two optic neuropathies.

Segmentation of the retina helps clinicians gain further insight into the detailed and precise pathophysiological mechanisms of the disease. The primary differences between MS and NMOSD in retinal structure and microvasculature lie in pRNFL, GC‐IPL, VD, and PD metrics, especially in the inferior and superior quadrants. This may be associated with the fact that the arcuate fibers (located in the superior and inferior quadrants) are commonly injured in vascular optic neuropathies. (Green & Cree, 2009) The central retinal artery extends with larger arteries and arterioles through the entire RNFL and ganglion cell layer and supplies the superficial vascular plexus. (Campbell et al., 2017) In addition, the differences between MS and NMOSD were concentrated in six quadrants of the GC‐IPL, which may be primarily due to the AQP4‐antibody‐damaged ganglion cells.

Consistent with a recent study(Murphy et al., 2020), we found significant differences in the microvascular parameters but no difference in the structural parameters when comparing MS‐ON with HCs, indicating that retinal vasculopathy may have existed in MS prior to structural atrophy. This might lend support to the important role of hypoperfusion in MS‐related damage. However, no difference was found between NMOSD‐ON and HCs in vascular comparison, which was inconsistent with previous studies showing subclinical primary retinal pathology in macular foveal thickness prior to pRNFL thinning. (Jeong et al., 2016; Pisa et al., 2019) The optic nerve of patients in the NMOSD + ON group is often affected near the chiasm, and potential carryover effects may impact the retina of the contralateral fellow eye after unilateral ON. (Ramanathan et al., 2016) We did not make any further subdivision due to the small number of NMOSD + ON eyes; therefore, the early vascular change of NMOSD‐ON was not accurately captured.

Furthermore, the presence of a primary retinopathy in AQP4‐IgG‐seropositive NMOSD, mediated by AQP4‐IgG, could be a pathophysiologic explanation for the observed changes. The bodies of Müller cells reside in the INL and process stretch through the whole thickness of the retina, connecting photoreceptors and retinal neurons with blood vessels. In contrast, AQP4 is expressed in the Müller cells’ end‐feet at the blood‐retina barrier. (Nagelhus & Ottersen, 2013) AQP4‐IgG may play an etiological role in vascular remodeling in NMOSD patients and may lead to cell loss or damage. (Bringmann et al., 2006) Interestingly, retinal vascular alterations have been reported in vivo, and pathologic studies have identified prominent vascular fibrosis and hyalinization in NMOSD lesions. (Green & Cree, 2009; Lucchinetti et al., 2002) OCTA analysis has revealed significant differences in the vascularization of the fovea have been found in patients with NMOSD in comparison with HCs. (Huang et al., 2019; Kwapong et al., 2018) This was consistent with the findings of our study, which went a step further and compared NMOSD and MS.

The size and shape of the FAZ have both been used as an outcome in diabetes mellitus. (Lu et al., 2018) However, in our study, no significance of FAZ was found, in comparison either between the patient group and HCs or between the patient groups.

We found that OCTA is better than OCT for the identification of the MS/NMOSD groups and MS + ON/NMOSD + ON subgroups. In addition, combined OCTA and OCT also had a better discrimination ability than OCT alone.

We acknowledge some limitations in our current study. First, our study only included patients with AQP4‐IgG‐positive NMOSD. Therefore, MOG‐IgG‐seropositive still exists but AQP4‐IgG‐seronegative and AQP4‐IgG and MOG‐Ig double‐negative cases manifesting clinical and neuroimaging signs of NMOSD. (Bruijstens et al., 2020; Zamvil & Slavin, 2015) Future studies should further investigate this point. Second, our study was retrospective in nature. Therefore, no analysis on the effect of treatment strategies was conducted.

In summary, our study compared the structure and flow between patients with MS and those with AQP4‐IgG‐positive NMOSD, indicating pathophysiological differences between them and suggesting that OCTA may have an additive effect as a biomarker in conjunction with routine OCT measurements. Combining OCTA and OCT may yield promising diagnostic properties in distinguishing both diseases. Further clinical trials are warranted to assess the potential implications of the retinal vascular parameters for the diagnosis and assessment of these disorders.

## CONFLICT OF INTEREST

The authors have no proprietary or commercial interest in any materials discussed in this article.

## AUTHOR CONTRIBUTIONS

Chunxin Liu, Hui Xiao, and Xiayin Zhang have contributed equally to the study. Wei Qiu and Haotian Lin: Conception and design. Chunxin Liu and Caixia Li: Analysis and interpretation. Chunxin Liu, Hui Xiao, Xiayin Zhang, Yipeng Zhao, Rui Li, Xiaonan Zhong, Yuge Wang, Yaqing Shu, Yanyu Chang, and Jingqi Wang: Data collection. Wei Qiu and Haotian Lin: Critically revised the manuscript. Wei Qiu: Obtained funding. Wei Qiu and Haotian Lin: Overall responsibility.

### PEER REVIEW

The peer review history for this article is available at https://publons.com/publon/10.1002/brb3.2125.

## Supporting information

Supplementary Material

Supplementary Material

Supplementary Material

Supplementary Material

Supplementary Material

## Data Availability

The data that support the findings of this study are available from the corresponding author upon reasonable request.

## References

[brb32125-bib-0001] Akaishi, T. , Takahashi, T. , Misu, T. , Abe, M. , Ishii, T. , Fujimori, J. , Aoki, M. , Fujihara, K. , Nakashima, I. (2020). Progressive patterns of neurological disability in multiple sclerosis and neuromyelitis optica spectrum disorders. Scientific Reports, 10(1), 13890. 10.1038/s41598-020-70919-w 32807848 PMC7431838

[brb32125-bib-0002] Alber, J. , Goldfarb, D. , Thompson, L. I. , Arthur, E. , Hernandez, K. , Cheng, D. , DeBuc, D. C. , Cordeiro, F. , Provetti‐Cunha, L. , den Haan, J. , Van Stavern, G. P. , Salloway, S. P. , Sinoff, S. & Snyder, P. J. (2020). Developing retinal biomarkers for the earliest stages of Alzheimer's disease: What we know, what we don't, and how to move forward. Alzheimer's & Dementia: The Journal of the Alzheimer's Association, 16(1), 229–243. 10.1002/alz.12006 31914225

[brb32125-bib-0003] Ayrignac, X. , Daliere, C. C. , Nerrant, E. , Vincent, T. , De Seze, J. , & Labauge, P. (2014). Extensive cerebral white matter involvement in a patient with NMO spectrum disorder. Multiple Sclerosis (Houndmills, Basingstoke, England), 20(10), 1401–1403. 10.1177/1352458514536253 24852925

[brb32125-bib-0004] Bennett, J. L. , de Seze, J. , Lana‐Peixoto, M. , Palace, J. , Waldman, A. , Schippling, S. , Tenembaum, S. , Banwell, B. , Greenberg, B. , Levy, M. , Fujihara, K. , Chan, K. H. , Kim, H. J. , Asgari, N. , Sato, D. K. , Saiz, A. , Wuerfel, J. , Zimmermann, H. , Green, A. , … Paul, F. (2015). Neuromyelitis optica and multiple sclerosis: Seeing differences through optical coherence tomography. Multiple Sclerosis (Houndmills, Basingstoke, England), 21(6), 678–688. 10.1177/1352458514567216 PMC4425816 25662342

[brb32125-bib-0005] Bringmann, A. , Pannicke, T. , Grosche, J. , Francke, M. , Wiedemann, P. , Skatchkov, S. N. , Osborne, N. N. , & Reichenbach, A. (2006). Muller cells in the healthy and diseased retina. Progress in Retinal and Eye Research, 25(4), 397–424. 10.1016/j.preteyeres.2006.05.003 16839797

[brb32125-bib-0006] Browne, P. , Chandraratna, D. , Angood, C. , Tremlett, H. , Baker, C. , Taylor, B. V. , & Thompson, A. J. (2014). Atlas of multiple sclerosis 2013: A growing global problem with widespread inequity. Neurology, 83(11), 1022–1024. 10.1212/WNL.0000000000000768 25200713 PMC4162299

[brb32125-bib-0007] Bruijstens, A. L. , Wong, Y. Y. M. , van Pelt, D. E. , van der Linden, P. J. E. , Haasnoot, G. W. , Hintzen, R. Q. , Claas, F. H. J. , Neuteboom, R. F. , & Wokke, B. H. A. (2020). HLA association in MOG‐IgG‐ and AQP4‐IgG‐related disorders of the CNS in the Dutch population. Neurology ‐ Neuroimmunology Neuroinflammation, 7(3), e702. 10.1212/NXI.0000000000000702 32198229 PMC7136059

[brb32125-bib-0008] Calabresi, P. A. , Balcer, L. J. , & Frohman, E. M. (2010). Retinal pathology in multiple sclerosis: Insight into the mechanisms of neuronal pathology. Brain, 133(Pt 6), 1575–1577. 10.1093/brain/awq133 20511281 PMC2877908

[brb32125-bib-0009] Campbell, J. P. , Zhang, M. , Hwang, T. S. , Bailey, S. T. , Wilson, D. J. , Jia, Y. , & Huang, D. (2017). Detailed vascular anatomy of the human retina by projection‐resolved optical coherence tomography Angiography. Scientific Reports, 7, 42201. 10.1038/srep42201 28186181 PMC5301488

[brb32125-bib-0010] Cruz‐Herranz, A. , Balk, L. J. , Oberwahrenbrock, T. , Saidha, S. , Martinez‐Lapiscina, E. H. , Lagreze, W. A. , Schuman, J. S. , Villoslada, P. , Calabresi, P. , Balcer, L. , Petzold, A. , Green, A. J. , Paul, F. , Brandt, A. U. , & Albrecht, P. (2016). The APOSTEL recommendations for reporting quantitative optical coherence tomography studies. Neurology, 86(24), 2303–2309. 10.1212/WNL.0000000000002774 27225223 PMC4909557

[brb32125-bib-0011] DeLong, E. R. , DeLong, D. M. , & Clarke‐Pearson, D. L. (1988). Comparing the areas under two or more correlated receiver operating characteristic curves: A nonparametric approach. Biometrics, 44(3), 837–845. 10.2307/2531595 3203132

[brb32125-bib-0012] Dogan, U. , Ulas, F. , Turkoglu, S. A. , Ogun, M. N. , & Agca, S. (2019). Eyes are mirror of the brain: Comparison of multiple sclerosis patients and healthy controls using OCT. International Journal of Neuroscience, 129(9), 848–855. 10.1080/00207454.2019.1576660 30696321

[brb32125-bib-0013] Fernandes, D. B. , Raza, A. S. , Nogueira, R. G. , Wang, D. , Callegaro, D. , Hood, D. C. , & Monteiro, M. L. (2013). Evaluation of inner retinal layers in patients with multiple sclerosis or neuromyelitis optica using optical coherence tomography. Ophthalmology, 120(2), 387–394. 10.1016/j.ophtha.2012.07.066 23084127 PMC3554837

[brb32125-bib-0014] Filippatou, A. G. , Mukharesh, L. , Saidha, S. , Calabresi, P. A. , & Sotirchos, E. S. (2020). AQP4‐IgG and MOG‐IgG related optic neuritis‐prevalence, optical coherence tomography findings, and visual outcomes: A systematic review and meta‐analysis. Frontiers in Neurology, 11, 540156. 10.3389/fneur.2020.540156 33132999 PMC7578376

[brb32125-bib-0015] Gabilondo, I. , Martínez‐Lapiscina, E. H. , Fraga‐Pumar, E. , Ortiz‐Perez, S. , Torres‐Torres, R. , Andorra, M. , Llufriu, S. , Zubizarreta, I. , Saiz, A. , Sanchez‐Dalmau, B. , & Villoslada, P. (2015). Dynamics of retinal injury after acute optic neuritis. Annals of Neurology, 77(3), 517–528. 10.1002/ana.24351 25559267

[brb32125-bib-0016] Green, A. J. & Cree, B. A. (2009). Distinctive retinal nerve fibre layer and vascular changes in neuromyelitis optica following optic neuritis. Journal of Neurology, Neurosurgery and Psychiatry, 80(9), 1002–1005. 10.1136/jnnp.2008.166207 19465415

[brb32125-bib-0017] Hokari, M. , Yokoseki, A. , Arakawa, M. , Saji, E. , Yanagawa, K. , Yanagimura, F. , Toyoshima, Y. , Okamoto, K. , Ueki, S. , Hatase, T. , Ohashi, R. , Fukuchi, T. , Akazawa, K. , Yamada, M. , Kakita, A. , Takahashi, H. , Nishizawa, M. , & Kawachi, I. (2016). Clinicopathological features in anterior visual pathway in neuromyelitis optica. Annals of Neurology, 79(4), 605–624. 10.1002/ana.24608 26836302

[brb32125-bib-0018] Huang, Y. , Zhou, L. , ZhangBao, J. , Cai, T. , Wang, B. , Li, X. , Wang, L. , Lu, C. , Zhao, C. , Lu, J. , Quan, C. , & Wang, M. (2019). Peripapillary and parafoveal vascular network assessment by optical coherence tomography angiography in aquaporin‐4 antibody‐positive neuromyelitis optica spectrum disorders. British Journal of Ophthalmology, 103(6), 789–796. 10.1136/bjophthalmol-2018-312231 PMC6582722 30021816

[brb32125-bib-0019] Jarius, S. , Paul, F. , Weinshenker, B. G. , Levy, M. , Kim, H. J. , & Wildemann, B. (2020). Neuromyelitis optica. Nature Reviews Disease Primers, 6(1), 85. 10.1038/s41572-020-0214-9 33093467

[brb32125-bib-0020] Jeong, I. H. , Kim, H. J. , Kim, N. H. , Jeong, K. S. , & Park, C. Y. (2016). Subclinical primary retinal pathology in neuromyelitis optica spectrum disorder. Journal of Neurology, 263(7), 1343–1348. 10.1007/s00415-016-8138-8 27142716

[brb32125-bib-0021] Kawachi, I. & Lassmann, H. (2017). Neurodegeneration in multiple sclerosis and neuromyelitis optica. Journal of Neurology, Neurosurgery and Psychiatry, 88(2), 137–145. 10.1136/jnnp-2016-313300 27671902

[brb32125-bib-0022] Kleerekooper, I. , Houston, S. , Dubis, A. M. , Trip, S. A. , & Petzold, A. (2020). Optical coherence tomography angiography (OCTA) in multiple sclerosis and neuromyelitis optica spectrum disorder. Frontiers in Neurology, 11, 604049. 10.3389/fneur.2020.604049 33362705 PMC7758345

[brb32125-bib-0023] Kwapong, W. R. , Peng, C. , He, Z. , Zhuang, X. , Shen, M. , & Lu, F. (2018). Altered macular microvasculature in neuromyelitis optica spectrum disorders. American Journal of Ophthalmology, 192, 47–55. 10.1016/j.ajo.2018.04.026 29750948

[brb32125-bib-0024] Lange, A. P. , Sadjadi, R. , Zhu, F. , Alkabie, S. , Costello, F. , & Traboulsee, A. L. (2013). Spectral‐domain optical coherence tomography of retinal nerve fiber layer thickness in NMO patients. Journal of Neuro‐Ophthalmology, 33(3), 213–219. 10.1097/WNO.0b013e31829c510e 23863782

[brb32125-bib-0025] Lu, Y. , Simonett, J. M. , Wang, J. , Zhang, M. , Hwang, T. , Hagag, A. M. , Huang, D. , Li, D. , & Jia, Y. (2018). Evaluation of automatically quantified foveal avascular zone metrics for diagnosis of diabetic retinopathy using optical coherence tomography angiography. Investigative Ophthalmology & Visual Science, 59(6), 2212–2221. 10.1167/iovs.17-23498 29715365 PMC5958306

[brb32125-bib-0026] Lucchinetti, C. F. , Mandler, R. N. , McGavern, D. , Bruck, W. , Gleich, G. , Ransohoff, R. M. , Trebst, C. , Weinshenker, B. , Wingerchuk, D. , Parisi, J. E. , & Lassmann, H. (2002). A role for humoral mechanisms in the pathogenesis of Devic's neuromyelitis optica. Brain, 125(Pt 7), 1450–1461. 10.1093/brain/awf151 12076996 PMC5444467

[brb32125-bib-0027] Merle, H. , Olindo, S. , Donnio, A. , Richer, R. , Smadja, D. , & Cabre, P. (2008). Retinal peripapillary nerve fiber layer thickness in neuromyelitis optica. Investigative Ophthalmology & Visual Science, 49(10), 4412–4417. 10.1167/iovs.08-1815 18614811

[brb32125-bib-0028] Motamedi, S. , Oertel, F. C. , Yadav, S. K. , Kadas, E. M. , Weise, M. , Havla, J. , Ringelstein, M. , Aktas, O. , Albrecht, P. , Ruprecht, K. , Bellmann‐Strobl, J. , Zimmermann, H. G. , Paul, F. , & Brandt, A. U. (2020). Altered fovea in AQP4‐IgG‐seropositive neuromyelitis optica spectrum disorders. Neurology ‐ Neuroimmunology Neuroinflammation, 7(5), 10.1212/NXI.0000000000000805 PMC7413713 32576604

[brb32125-bib-0029] Murphy, O. C. , Kwakyi, O. , Iftikhar, M. , Zafar, S. , Lambe, J. , Pellegrini, N. , Sotirchos, E. S. , Gonzalez‐Caldito, N. , Ogbuokiri, E. , Filippatou, A. , Risher, H. , Cowley, N. , Feldman, S. , Fioravante, N. , Frohman, E. M. , Frohman, T. C. , Balcer, L. J. , Prince, J. L. , Channa, R. , … Saidha, S. (2020). Alterations in the retinal vasculature occur in multiple sclerosis and exhibit novel correlations with disability and visual function measures. Multiple Sclerosis (Houndmills, Basingstoke, England), 26(7), 815–828. 10.1177/1352458519845116 PMC6858526 31094280

[brb32125-bib-0030] Nagelhus, E. A. & Ottersen, O. P. (2013). Physiological roles of aquaporin‐4 in brain. Physiological Reviews, 93(4), 1543–1562. 10.1152/physrev.00011.2013 24137016 PMC3858210

[brb32125-bib-0031] Oertel, F. C. , Zimmermann, H. , Mikolajczak, J. , Weinhold, M. , Kadas, E. M. , Oberwahrenbrock, T. , Pache, F. , Bellmann‐Strobl, J. , Ruprecht, K. , Paul, F. , & Brandt, A. U. (2017). Contribution of blood vessels to retinal nerve fiber layer thickness in NMOSD. Neurology ‐ Neuroimmunology Neuroinflammation, 4(3), e338. 10.1212/NXI.0000000000000338 28451626 PMC5398419

[brb32125-bib-0032] Oertel, F. C. , Zimmermann, H. , Paul, F. , & Brandt, A. U. (2018). Optical coherence tomography in neuromyelitis optica spectrum disorders: Potential advantages for individualized monitoring of progression and therapy. EPMA Journal, 9(1), 21–33. 10.1007/s13167-017-0123-5 PMC5833887 29515685

[brb32125-bib-0033] Oh, J. , Sotirchos, E. S. , Saidha, S. , Whetstone, A. , Chen, M. , Newsome, S. D. Zackowski, K. , Balcer, L. J. , Frohman, E. , Prince, J. , Diener‐West, M. , Reich, D. S. , & Calabresi, P. A. (2015). Relationships between quantitative spinal cord MRI and retinal layers in multiple sclerosis. Neurology, 84(7), 720–728. 10.1212/WNL.0000000000001257 25609766 PMC4336102

[brb32125-bib-0034] Ohayon, A. , Sacconi, R. , Semoun, O. , Corbelli, E. , Souied, E. H. , & Querques, G. (2020). Choroidal neovascular area and vessel density comparison between two swept‐source optical coherence tomography angiography devices. Retina, 40(3), 521–528. 10.1097/IAE.0000000000002430 30589664

[brb32125-bib-0035] Pisa, M. , Ratti, F. , Vabanesi, M. , Radaelli, M. , Guerrieri, S. , Moiola, L. , Martinelli, V. , Comi, G. , & Leocani, L. (2019). Subclinical neurodegeneration in multiple sclerosis and neuromyelitis optica spectrum disorder revealed by optical coherence tomography. Multiple Sclerosis (Houndmills, Basingstoke, England), 26(10), 1197–1206. 10.1177/1352458519861603 31392924

[brb32125-bib-0036] Ramanathan, S. , Prelog, K. , Barnes, E. H. , Tantsis, E. M. , Reddel, S. W. , Henderson, A. P. D. , Vucic, S. , Gorman, M. P. , Benson, L. A. , Alper, G. , Riney, C. J. , Barnett, M. , Parratt, J. D. E. , Hardy, T. A. , Leventer, R. J. , Merheb, V. , Nosadini, M. , Fung, V. S. C. , Brilot, F. , … Dale, R. C. (2016). Radiological differentiation of optic neuritis with myelin oligodendrocyte glycoprotein antibodies, aquaporin‐4 antibodies, and multiple sclerosis. Multiple Sclerosis (Houndmills, Basingstoke, England), 22(4), 470–482. 10.1177/1352458515593406 26163068

[brb32125-bib-0037] Rebolleda, G. , Sanchez‐Sanchez, C. , Gonzalez‐Lopez, J. J. , Contreras, I. , & Munoz‐Negrete, F. J. (2015). Papillomacular bundle and inner retinal thicknesses correlate with visual acuity in nonarteritic anterior ischemic optic neuropathy. Investigative Ophthalmology & Visual Science, 56(2), 682–692. 10.1167/iovs.14-15314 25587057

[brb32125-bib-0038] Saidha, S. , Sotirchos, E. S. , Oh, J. , Syc, S. B. , Seigo, M. A. , Shiee, N. , Eckstein, C. , Durbin, M. K. , Oakley, J. D. , Meyer, S. A. , Frohman, T. C. , Newsome, S. T. , Ratchford, J. N. , Balcer, L. J. , Pham, D. L. , Crainiceanu, C. M. , Frohman, E. M. , Reich, D. S. , & Calabresi, P. A. (2013). Relationships between retinal axonal and neuronal measures and global central nervous system pathology in multiple sclerosis. JAMA Neurology, 70(1), 34–43. 10.1001/jamaneurol.2013.573 23318513 PMC4030557

[brb32125-bib-0039] Schippling, S. , Balk, L. J. , Costello, F. , Albrecht, P. , Balcer, L. , Calabresi, P. A. , Frederiksen, J. L. , Frohman, E. , Green, A. J. , Klistorner, A. , Outteryck, O. , Paul, F. , Plant, G. T. , Traber, G. , Vermersch, P. , Villoslada, P. , Wolf, S. , & Petzold, A. (2015). Quality control for retinal OCT in multiple sclerosis: Validation of the OSCAR‐IB criteria. Multiple Sclerosis (Houndmills, Basingstoke, England), 21(2), 163–170. 10.1177/1352458514538110 24948688

[brb32125-bib-0040] Shen, T. , You, Y. , Arunachalam, S. , Fontes, A. , Liu, S. , Gupta, V. , Parratt, J. , Wang, C. , Barnett, M. , Barton, J. , Chitranshi, N. , Zhu, L. , Fraser, C. L. , Graham, S. L. , Klistorner, A. , & Yiannikas, C. (2019). Differing structural and functional patterns of optic nerve damage in multiple sclerosis and neuromyelitis optica spectrum disorder. Ophthalmology, 126(3), 445–453. 10.1016/j.ophtha.2018.06.022 30060979

[brb32125-bib-0041] Sotirchos, E. S. , Filippatou, A. , Fitzgerald, K. C. , Salama, S. , Pardo, S. , Wang, J. , Ogbuokiri, E. , Cowley, N. J. , Pellegrini, N. , Murphy, O. C. , Mealy, M. A. , Prince, J. L. , Levy, M. , Calabresi, P. A. , & Saidha, S. (2020). Aquaporin‐4 IgG seropositivity is associated with worse visual outcomes after optic neuritis than MOG‐IgG seropositivity and multiple sclerosis, independent of macular ganglion cell layer thinning. Multiple Sclerosis (Houndmills, Basingstoke, England), 26(11), 1360–1371. 10.1177/1352458519864928 PMC6992495 31364464

[brb32125-bib-0042] Spain, R. I. , Liu, L. , Zhang, X. , Jia, Y. , Tan, O. , Bourdette, D. , & Huang, D. (2018). Optical coherence tomography angiography enhances the detection of optic nerve damage in multiple sclerosis. British Journal of Ophthalmology, 102(4), 520–524. 10.1136/bjophthalmol-2017-310477 PMC6467481 28814415

[brb32125-bib-0043] Tavazzi, E. , Jakimovski, D. , Kuhle, J. , Hagemeier, J. , Ozel, O. , Ramanathan, M. , Barro, C. , Bergsland, N. , Tomic, D. , Kropshofer, H. , Leppert, D. , Michalak, Z. , Lincoff, N. , Dwyer, M. G. , Benedict, R. H. B. , Weinstock‐Guttman, B. , & Zivadinov, R. (2020). Serum neurofilament light chain and optical coherence tomography measures in MS: A longitudinal study. Neurology ‐ Neuroimmunology Neuroinflammation, 7(4), 10.1212/NXI.0000000000000737 PMC7251512 32424064

[brb32125-bib-0044] Thompson, A. J. , Banwell, B. L. , Barkhof, F. , Carroll, W. M. , Coetzee, T. , Comi, G. , & Cohen, J. A. (2018). Diagnosis of multiple sclerosis: 2017 revisions of the McDonald criteria. The Lancet Neurology, 17(2), 162–173. 10.1016/S1474-4422(17)30470-2 29275977

[brb32125-bib-0045] Weinshenker, B. G. & Wingerchuk, D. M. (2017). Neuromyelitis spectrum disorders. Mayo Clinic Proceedings, 92(4), 663–679. 10.1016/j.mayocp.2016.12.014 28385199

[brb32125-bib-0046] Wingerchuk, D. M. , Banwell, B. , Bennett, J. L. , Cabre, P. , Carroll, W. , Chitnis, T. , de Seze, J. , Fujihara, K. , Greenberg, B. , Jacob, A. , Jarius, S. , Lana‐Peixoto, M. , Levy, M. , Simon, J. H. , Tenembaum, S. , Traboulsee, A. L. , Waters, P. , Wellik, K. E. , & Weinshenker, B. G. (2015). International consensus diagnostic criteria for neuromyelitis optica spectrum disorders. Neurology, 85(2), 177–189. 10.1212/WNL.0000000000001729 26092914 PMC4515040

[brb32125-bib-0047] Yan, J. (2002). geepack: Yet another package for generalized estimating equations. R‐News, 2(3), 12–14.

[brb32125-bib-0048] You, Y. , Barnett, M. H. , Yiannikas, C. , Parratt, J. , Matthews, J. , Graham, S. L. , & Klistorner, A. (2020). Chronic demyelination exacerbates neuroaxonal loss in patients with MS with unilateral optic neuritis. Neurology ‐ Neuroimmunology Neuroinflammation, 7(3), 10.1212/NXI.0000000000000700 PMC7136042 32170043

[brb32125-bib-0049] Zamvil, S. S. & Slavin, A. J. (2015). Does MOG Ig‐positive AQP4‐seronegative opticospinal inflammatory disease justify a diagnosis of NMO spectrum disorder? Neurol Neuroimmunol Neuroinflamm, 2(1), e62. 10.1212/NXI.0000000000000062 25635259 PMC4309526

[brb32125-bib-0050] Zhang, X. , Xiao, H. , Liu, C. , Liu, S. , Zhao, L. , Wang, R. , Wang, J. , Wang, T. , Zhu, Y. , Chen, C. , Wu, X. , Lin, D. , Qiu, W. , Yu‐Wai‐Man, P. , Lu, Z. , & Lin, H. (2020). Optical coherence tomography angiography reveals distinct retinal structural and microvascular abnormalities in cerebrovascular disease. Frontiers in Neuroscience, 14, 588515. 10.3389/fnins.2020.588515 33132836 PMC7561709

[brb32125-bib-0051] Zhang, X. , Xiao, H. , Liu, C. , Zhao, L. , Wang, J. , Li, H. , Wang, R. , Zhu, Y. , Chen, C. , Wu, X. , Lin, D. , Wang, J. , Liu, X. , Qiu, W. , Yu‐Wai‐Man, P. , Ting, D. S. , & Lin, H. (2020). Comparison of macular structural and vascular changes in neuromyelitis optica spectrum disorder and primary open angle glaucoma: A cross‐sectional study. British Journal of Ophthalmology, 105(3), 354–360. 10.1136/bjophthalmol-2020-315842 PMC7907571 32430343

[brb32125-bib-0052] Zubizarreta, I. , Flórez‐Grau, G. , Vila, G. , Cabezón, R. , España, C. , Andorra, M. , & Villoslada, P. (2019). Immune tolerance in multiple sclerosis and neuromyelitis optica with peptide‐loaded tolerogenic dendritic cells in a phase 1b trial. Proceedings of the National Academy of Sciences USA, 116(17), 8463–8470. 10.1073/pnas.1820039116 PMC6486735 30962374

