# Supplementary figures and images for: Optical coherence tomography angiography helps distinguish multiple sclerosis from AQP4‐IgG‐seropositive neuromyelitis optica spectrum disorder
Source: Brain Behav. 2021 Mar 30;11(5):e02125. doi: 10.1002/brb3.2125 (PMC8119797; doi:10.1002/brb3.2125)

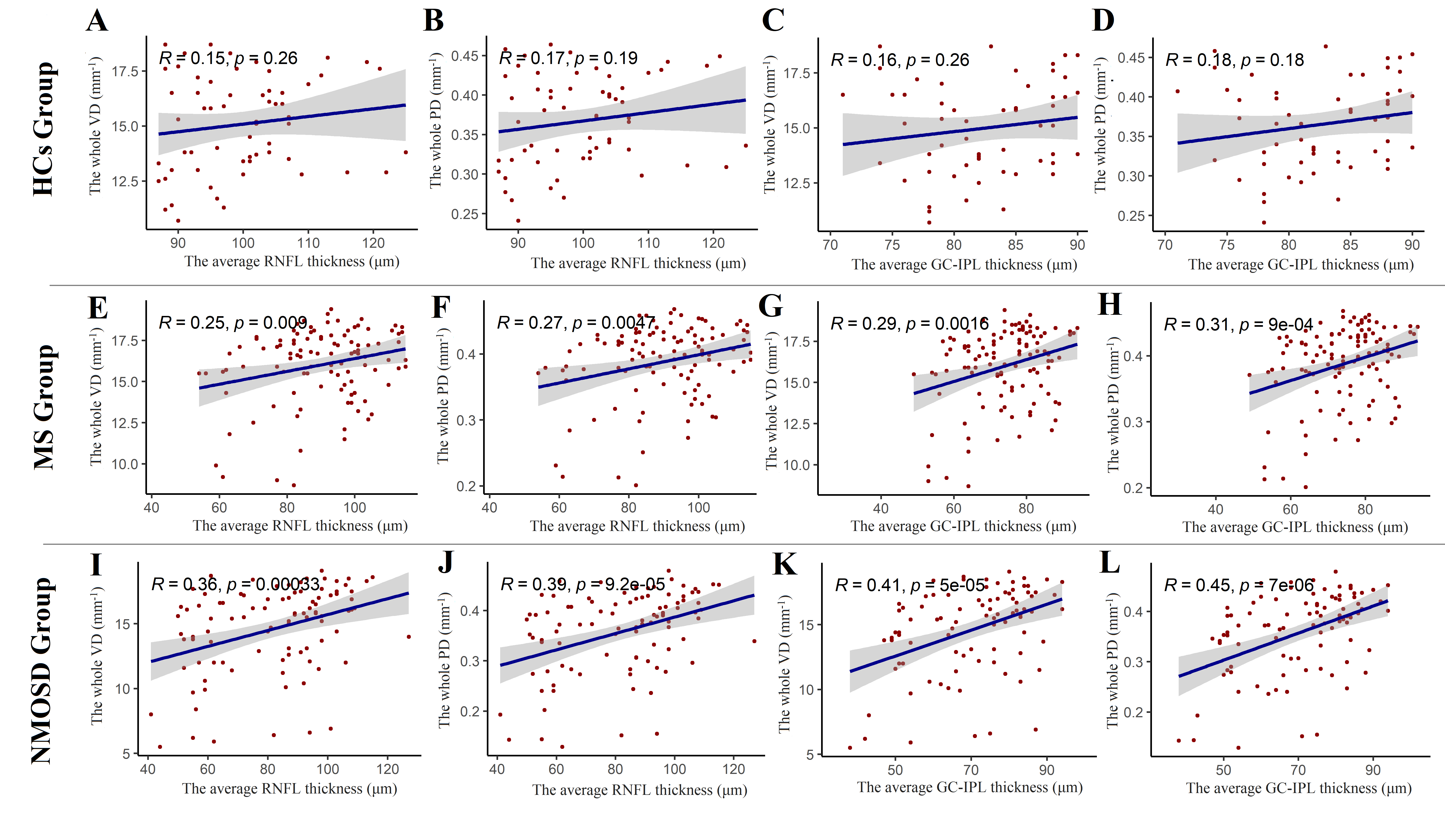

Supplement: Supplementary file 1 — Supplementary Material [file BRB3-11-e02125-s005.tif]

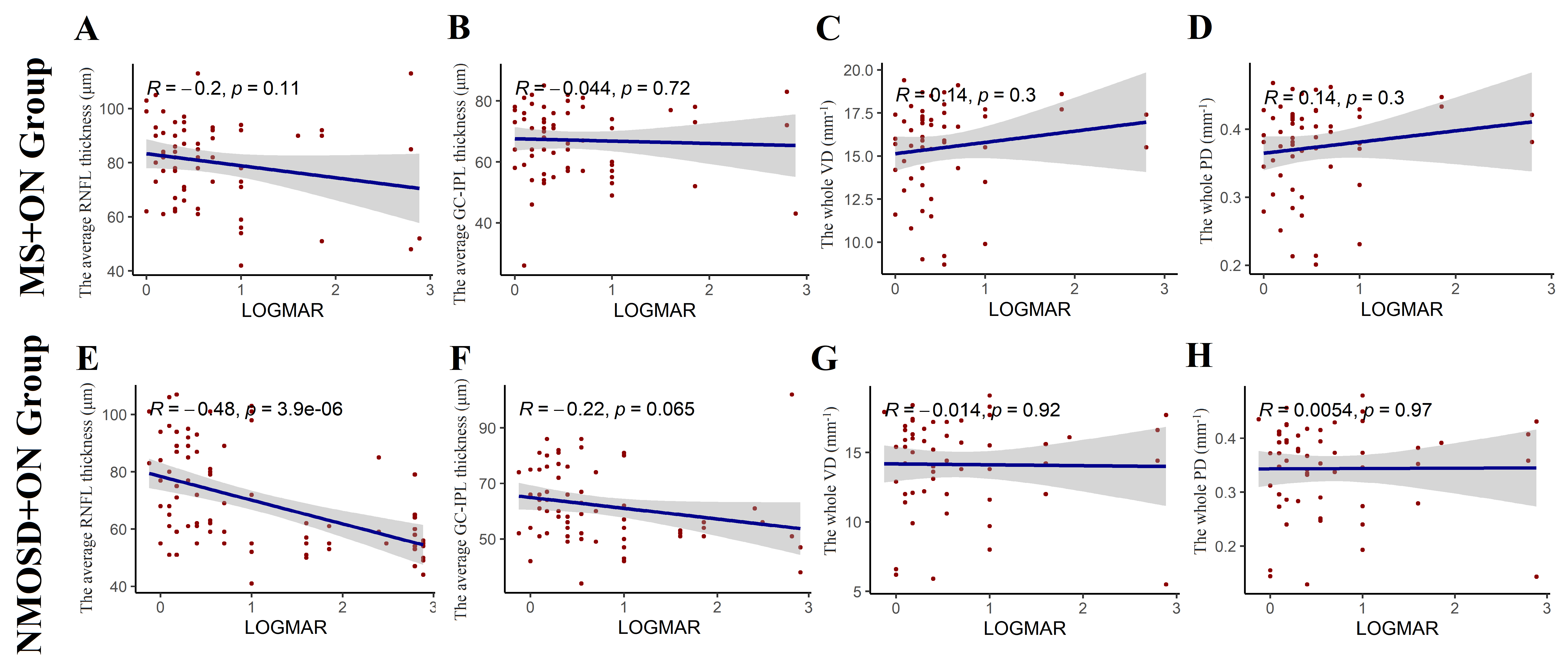

Supplement: Supplementary file 2 — Supplementary Material [file BRB3-11-e02125-s001.tif]

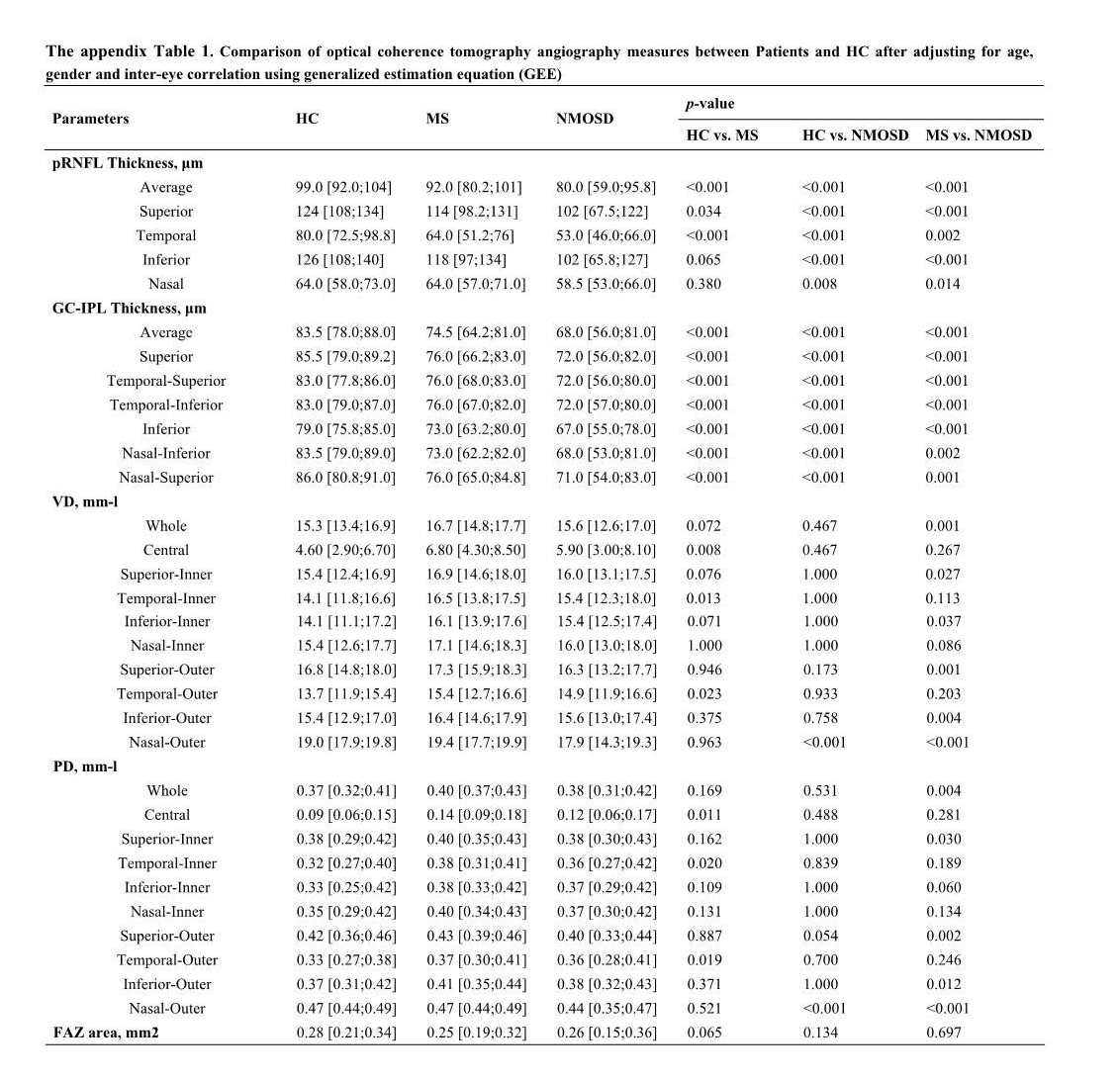

Supplement: Supplementary file 3 — Supplementary Material [file BRB3-11-e02125-s003.tif]

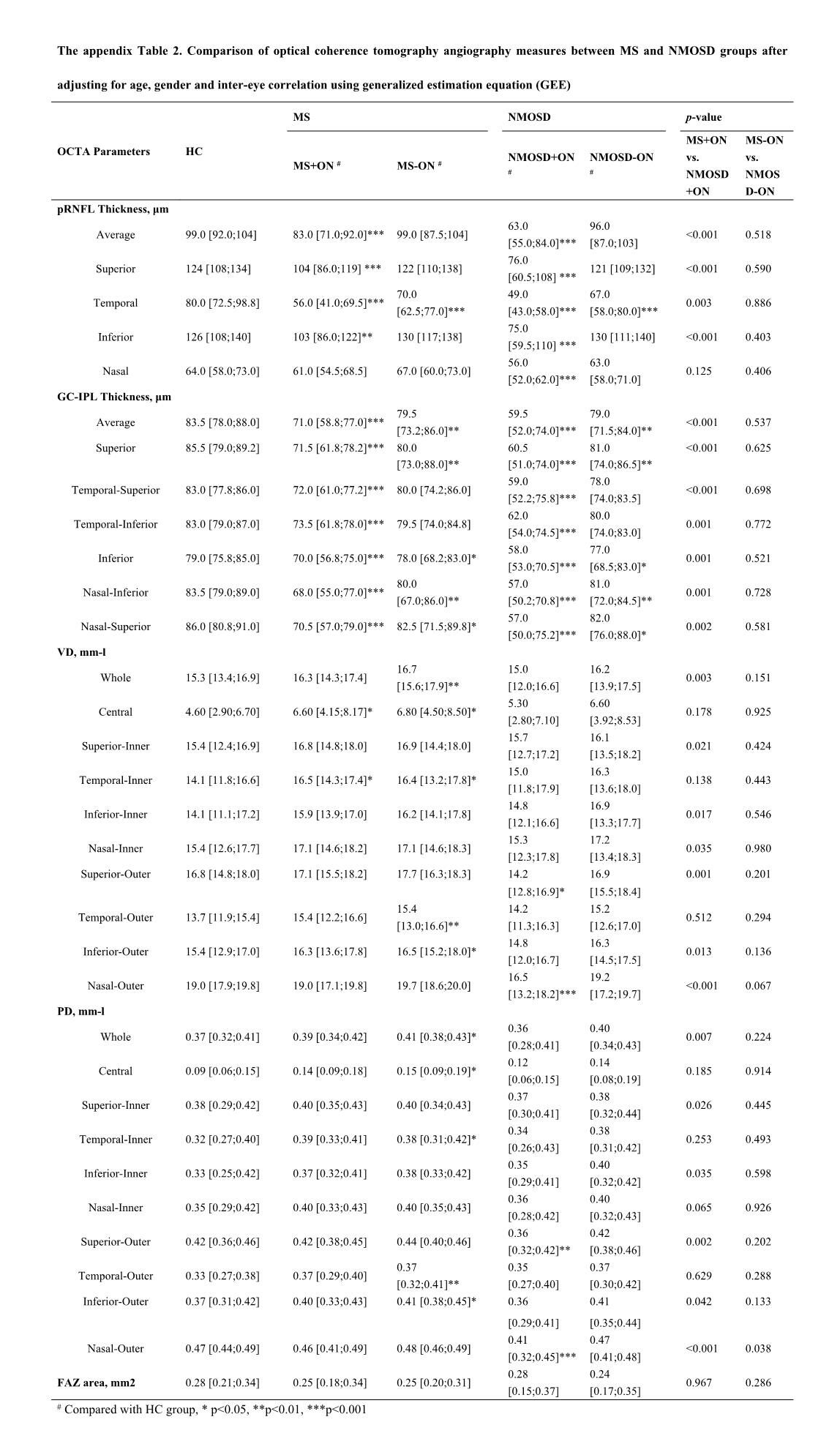

Supplement: Supplementary file 4 — Supplementary Material [file BRB3-11-e02125-s004.tif]

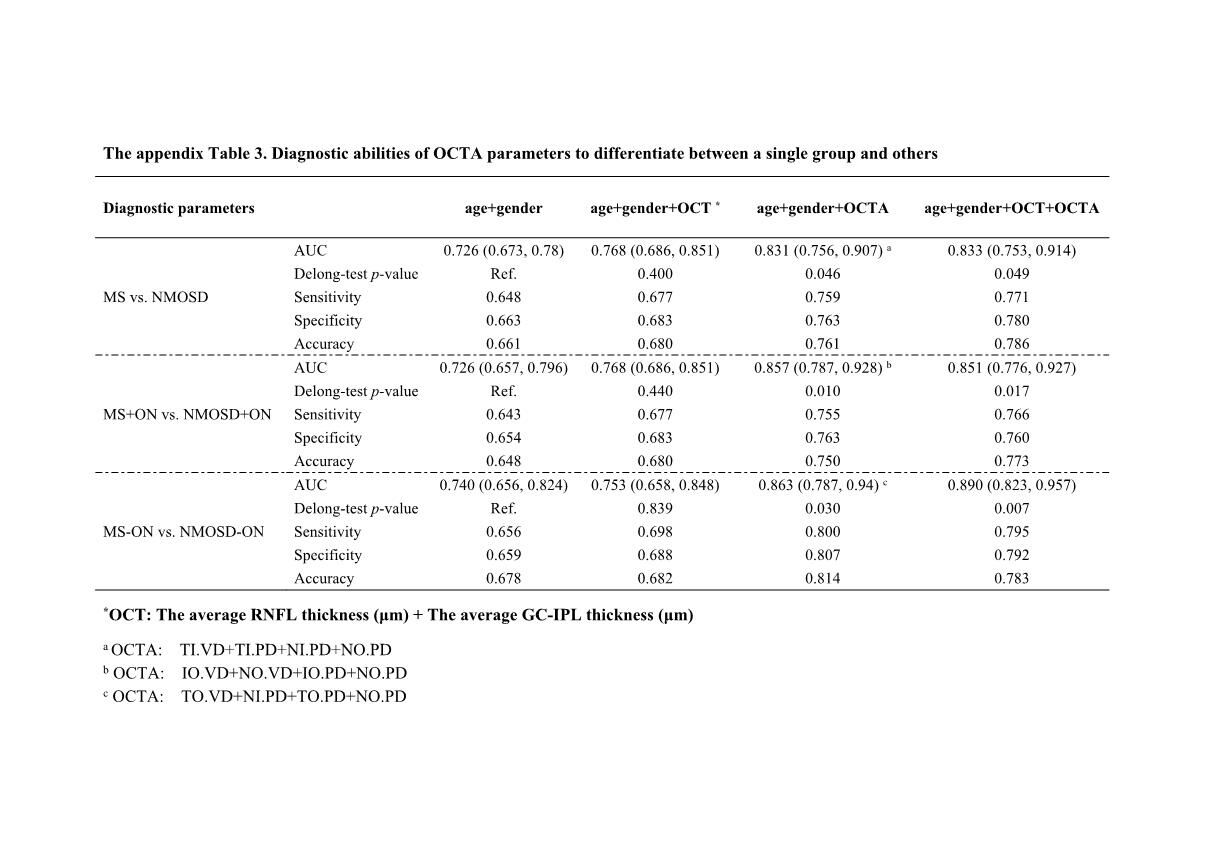

Supplement: Supplementary file 5 — Supplementary Material [file BRB3-11-e02125-s002.tif]
